# Factors influencing improved attendance in the UK fire service

**DOI:** 10.1093/occmed/kqw156

**Published:** 2016-11-03

**Authors:** I. Litchfield, P. Hinckley

**Affiliations:** ^1^Institute of Applied Health Research, College of Medical and Dental Sciences, University of Birmingham, Birmingham B15 2TT, UK,; ^2^West Midlands Fire Service, Headquarters, 99 Vauxhall Road, Birmingham B7 4HW, UK.

**Keywords:** Healthy workplaces, occupational health management, occupational stress, sickness absence, sick leave.

## Abstract

**Background:**

Sickness absence rates in the UK continue to exceed those in much of the developed world, with an annual cost to employers of £29 billion. Rates of sickness absence in the public sector are higher than those in the private sector, with the exception of the fire service where they are consistently lower.

**Aims:**

To understand the influences that increase attendance among operational firefighters.

**Methods:**

A series of semi-structured interviews undertaken with operational staff to explore their attitudes to sickness absence.

**Results:**

Review and analysis of participant responses identified a number of key themes, namely employee well-being, including physical fitness and mental health; employee engagement with the fire service as manifested by culture, experience, nature of the job and leadership; organizational factors including the staffing model and relationship with occupational health services and policy, which describes both refinements to and implementation of targeted policies.

**Conclusions:**

Previously observed factors such as improved fitness and the distinct firefighter culture play a role, yet other factors emerged that could explain the differences. These include the greater work–life balance offered by their shift patterns, the terms and conditions of employment and perhaps most importantly the evolution of precisely targeted policies that understand the unique nature of the operational fire service.

## Introduction

The rates of sickness absence in the UK, where workers take an average of 9.1 sick days per year, costing employers around £29 billion in sick pay and employees £4 billion in lost earnings [1], are of continued concern. Previous work has attempted to identify the influences at both individual [2] and organizational level [3] and a number of approaches have tried to reduce sickness absence [4]. Despite these attempts, the rate of sickness absence in the UK continues to be among the highest in the developed world particularly within the public sector [1]. There are a number of factors that might explain this difference; for example, public sector workers are proportionately more reliant on public transport than workers in the private sector or are women with poorer than average health [5]. However, the fire service is one public sector organization where rates of sickness absence more closely resemble those of the private sector [6]. Recent data gathered from the West Midlands Fire Service (WMFS) reflect this trend, most notably among operational firefighters where the 6.0 sick days taken annually compares favourably to the 7.3 days taken by their non-operational colleagues and the national average of 8.7 days across the public sector as a whole [7].

At first glance, these figures may appear incongruous considering the hazardous nature of operational firefighting and the risk of physical injury and exposure to psychological stress [8,9]. These lower rates may be attributable to higher levels of physical fitness or the distinct culture of operational firefighters, anchored by the socio-cultural identity of ‘firemen as heroes’ [10,11]. Yet the more precise identification of the influences that have led to a reduction in sickness absence in operational fire fighters may determine whether they can be used to inform strategies to improve attendance both among their non-operational colleagues and more broadly across the public sector. Here, we begin the process by exploring the views of operational staff on the factors that influence their attitudes toward sickness absence, including the relationship with co-workers and managers, perceptions of their role in the organization and the strategies used to manage sickness absence.

## Methods

The study took place within WMFS, the second largest UK Fire and Rescue Service (UKFRS) and one that provides emergency services to 2.7 million residents of the seven local authorities within the West Midlands metropolitan county. The workforce consists of some 1500 operational and 500 non-operational staff. Operational staff are made up of firefighters or fire officers, organized into groups known as ‘watches’, working either day or night shifts typically in a pattern of 4 days on and 4 days off. Non-operational staff work in fire control and are responsible for handling emergency calls to the service, with shift patterns similar to operational staff, or in specialist and administrative roles such as human resources or finance where staff typically work more conventional office hours.

All those invited to participate in the study were operational staff directly employed by WMFS and purposively sampled to reflect a range of rank, gender and length of service. The intention was not to sample proportionate numbers of each rank but instead for our sample to incorporate maximum variance, i.e. to reflect the range of seniority of operational employees. Written invitations were issued to all six operational watches from occupational health. Semi-structured interviews by the second author, a member of the occupational health unit at WMFS, were used to capture individual experiences of operational staff regarding their motivation to attend and the factors that influence this motivation. These include attitudes to their role and to their colleagues and the current management of sickness absence within the service (Box 1). Collection of data was to proceed until theoretical saturation was reached. All the interviews were digitally recorded and transcribed verbatim. The data were analysed using a constructivist grounded theory approach that understands researchers make consistent and ongoing subjective interpretations of the data grounded in their own perspectives, positions and interactions [12]. In analysing the data, the authors independently coded three transcripts from which broad categories of data or ‘themes’ were elucidated. We then met to discuss and define these and produce the final interpretative hierarchy of themes and sub-themes. The study was ethically approved by the University of Birmingham and WMFS.

**Box 1. Interview schedule**

1. What emphasis do you place on the importance of physical health?
2. What emphasis do you place on mental health?
3. Are there any aspects of your role that encourage you or your colleagues to attend work?
4. Are there any aspects of your work environment that encourage you or your colleagues to attend work?
5. Are there any aspects of the way your work is organized that encourage you or your colleagues to attend work?
6. What impact if any have management policies had on whether you or your colleagues attend work?

## Results

A total of 16 interviews were conducted with staff who had served between 8 and 28 years and ranged in rank from station commander to firefighter. The response rate was low at 24%, although the requisite job titles and watches were represented in the group interviewed. Only two of the participants were female which though small is indicative of the lower numbers of female operational staff. The characteristics of those interviewed are summarized in Table 1. All interviews were conducted face-to-face and their average length was 31 min. Theoretical saturation was reached after 16 interviews [13]. A total of four key themes and a number of associated sub-themes emerged; these key themes were employee health, employee engagement, organizational factors and policy. These are summarized alongside their sub-themes in Table 2 and described in detail below.

**Table 1.** Characteristics of interviewees

Job title	Gender	Length of service (years)
Station commander	Male	28
	Male	26
	Male	18
Group manager	Male	23
	Male	22
Watch manager	Male	27
	Male	26
	Male	9
	Male	8
Principal officer	Male	24
	Male	23
Crew commander	Female	10
Fire fighter	Male	14
	Male	12
	Female	11
	Male	9

**Table 2.** Thematic analysis

	Key theme	Sub-theme 1
1	Employee health	1.1 Physical fitness
		1.2 Mental health
2	Employee engagement	2.1 Masculine culture
		2.2 Nature of the job
		2.3 Experience
		2.4 Leadership
3	Organizational factors	3.1 Staffing model
4	Policy	4.1 Refined policy
		4.2 Implementation
		4.3 Financial incentives

The first theme of ‘employee health’ describes the attitudes of operational staff to physical fitness and mental health. This included how they related to exercise and their attitudes to mental health and the reluctance to use the word stress. Quotations illustrating these findings are contained in Table 3. Due to the physical nature of much of their work, operational staff were encouraged to exercise as part of their working day (quotation 1) and the extent of an individual’s physical fitness became a source of competition between some operational staff (quotation 2). However, this focus on physical fitness did not appear to be consistent across all grades of operational staff and some reported a decline in fitness as their seniority in rank increased (quotation 3). In discussing their mental health operational staff talked of the challenge of making appropriate decisions in difficult situations but appeared reluctant to describe the pressure they felt as ‘stress’ (quotation 4 and quotation 5).

**Table 3.** Illustrative quotations for the theme ‘employee health’

Quotation 1	‘Essentially firefighters have to be physically fit to do their roles, and should have the opportunity to do some form of physical fitness whilst in the workplace. Non-operational staff come from a different perspective. There may be components of age or disability in evidence, and we don’t monitor their levels of fitness in the same way …’ (station commander, male, 28 years service).
Quotation 2	‘There is a very competitive nature on stations in terms of fitness.’ (group manager, male, 23 years service).
Quotation 3	‘I was in my late twenties when I started in the fire service ... my overall health was pretty good right up until the time I became a station commander. I put nearly three stones on then.’ (group manager, male, 22 years service).
Quotation 4	‘When I come to work I understand the expectations of my role, and I sometimes have to make some challenging decisions, would I say it is stressful? No.’ (station commander, male, 26 years service).
Quotation 5	‘Taking charge of a ten-pump job at 2 o’clock in the morning, when it’s snowing, explosives going off all around you, and you’ve got responsibility for the safety of thirty, forty or fifty firefighters, that’s quite stressful. Actually, no—it’s not stressful—it’s *satisfyingly challenging.*’ (watch manager, male, 9 years service).

The second theme was ‘employee engagement’ which describes the connection staff felt with their colleagues and their organization (Table 4). It appeared that several felt that they shared characteristics with their colleagues as a result of the unique demands of the job and described the close-knit nature of their team as a powerful incentive to attend work. Many operational staff described how the nature of the job attracted similar individuals (quotation 6) and described a feeling of doing something out of the ordinary; for example, how their job varies from day-to-day in comparison to the perceived predictability of office jobs (quotation 7). The nature of the work and the shared characteristics of colleagues seemed an important incentive to attend work (quotation 8). However, it was felt that the close-knit nature of watch teams could have negative connotations if relationships had failed (quotation 9) or where the level of leadership was poor (quotation 10). This level of engagement did not appear to be static and participants described how personal attitudes to attendance had changed over time. One firefighter confessed that when younger they may have taken a day off without thinking of the consequences for colleagues or the organization (quotation 11). A watch manager also suggested that more senior staff are more likely to attend work, even if ill (quotation 12).

**Table 4.** Illustrative quotes describing the theme ‘employee engagement’

Quotation 6	‘It does take a certain state of mind to be able to place yourself in a situation that other people would like to run away from. I think that [firefighting] does require a certain type of individual.’ (fire fighter, male, 14 years service).
Quotation 7	‘A lot of people who aren’t operational attend a workplace where every day the work is the same, and mentally they get bored with consistently doing the same thing day in day out, and so the slightest reason not to attend work is a positive way for them to stop being bored, whereas with ourselves we attend work and for 90 per cent of the day we have no idea what we are going to be doing, and therefore every day is a reasonably exciting day.’ (fire fighter, male, 12 years service).
Quotation 8	‘There is a cultural belief or attitude amongst firefighters that makes them want to be at work, in the job. It’s about working as a team. If you have a close team, it doesn’t matter about the shift patterns that we work, or this, that, and the other, you enjoy coming to work and that feeling of bonding.’ (station commander, male, 28 years service).
Quotation 9	‘A good watch is an amazing place to be. A bad watch, maybe when you’ve got strong personalities that constantly disagree with one another, fight, or bicker ... In such a close-knit environment, relationships can be torn apart as easily as they can be nurtured. On a bad watch, if you’re off sick, the motivation to return to work would not be as great as if you were returning to a good watch.’ (watch manager, male, 9 years service).
Quotation 10	‘Leadership is the key. We’ve had a succession of gaffers who haven’t necessarily been consistent in their management. They’ve been both autocratic and laissez-faire. The whole watch at one point felt like they didn’t want to come to work. The effect was across the board.’ (crew commander, female, 10 years service).
Quotation 11	‘In the past, when I was younger, I thought to myself “I fancy a day off” maybe because I’m missing something and I could just phone in sick, because it doesn’t matter. They can’t sack me for it. So yes, I do believe that thought process does go round. I’ve since grown up and realised that was a mistake.’ (fire fighter, male, 9 years service).
Quotation 12	‘People who are more likely to be at work when clearly they shouldn’t be tend to be higher up the food chain, so it’s senior managers, station commanders and above, because they think that they have to set an example to the rest … and that’s why you never see the chief fire officer going sick, but they’re still human and they still get ill.’ (watch manager, male, 8 years service).

The third theme identified, ‘organizational factors’ describes the structure of the organization and how resources are utilized (Table 5). Specifically participants described the advantages of the shift model, particularly how the four on four off rota appeared to provide the opportunity for improved work–life balance and allowed the chance for firefighters to connect with their friends and family (quotations 13 and 14). The final theme was ‘Policy’ (Table 5), which describes the perceived impact of reconfigured policies, their implementation and the response of staff to financial incentives to attend work. One senior staff member commented on how attitudes to sickness absence have changed as the system for managing absence has improved (quotation 15). There was the belief that as sickness absence policies became more precise they helped eliminate the traditional assumption among firefighters of an entitlement to sick leave (quotation 16). One particular change to policy that was deemed effective was to replace a firefighter on sick leave with someone from his watch (quotation 17).

**Table 5.** Illustrative quotes describing the themes ‘organizational factors’ and ‘improved policy’

Quotation 13	‘I thoroughly enjoy my job. It is stressful and demanding, but I have family support and a decent work-life balance.’ (station commander, male, 28 years service).
Quotation 14	‘Having the four days off means I can plan things well in advance and arrange child care.’ (fire fighter, female, 11 years service)
Quotation 15	‘I just hope that all sickness nowadays is genuine, which hasn’t been the case in the past. There is a much more robust system now. Historically, sickness was abused and approached disrespectfully.’ (watch manager, male, 26 years service).
Quotation 16	‘‘‘Sickness entitlement” are words that were used in the Grey Book. You had a sickness “entitlement”, and what that meant was you were allowed *x* number of days before something was said about it, and that was used. I can remember my days on fire stations; there were people who would take their “entitlement” for sick leave every year, and they saw it as an entitlement to take that. I would hope that people don’t have that same approach and culture today.’ (principal officer, male, 23 years service).
Quotation 17	‘The new staffing model has reduced sickness absence in firefighters. Under the old system, if I went sick because I wanted a Saturday night off, they’d have to get a standby in from somewhere else, so it wouldn’t affect anyone on my watch. Now that it has a direct impact on others within my own watch, I think peer pressure has made a big difference.’ (station commander, male, 28 years service).
Quotation 18	‘CPD was the big one. People were worried about going past a certain level of sickness and losing their CPD. Those casual sick days that were thrown in don’t happen anymore; people want to leave a buffer just in case something does happen to them.’ (station commander, male, 28 years service).
Quotation 19	‘Whilst the attendance management policy has been very effective in reducing unnecessary absences, it also means that people have come to work when sick to avoid triggers.’ (station commander, male, 18 years service).
Quotation 20	‘I don’t think that it’s just the policies, but it’s the way the policies are implemented. There has been a lot more scrutiny and focus in the uniformed service on attendance levels, and over a period of time, a period of years, that has driven the supervisory managers and station commanders to a really clear understanding of how the policies work, what the options are to them, how they manage attendance back to work, what point they move on to the next stage, and whether to deal with that formally, informally and all the rest of it.’ (principal officer, male, 23 years service).

Financial incentives were also described and reference made to the continuing professional development (CPD) payment of up to £900 per annum available to operational individuals as part of their National Joint Council terms and conditions of employment. This payment can be withheld if sickness absence exceeds stated limits (quotation 18). However, potential drawbacks in the form of presenteeism were also recognized as one consequence of using these financial rewards (quotation 19). Whatever the policy, there was an understanding that they were only successful if implemented correctly and the importance of raising awareness in senior staff of the content of the policy and how it should be executed was noted (quotation 20).

## Discussion

A number of potential influences on attendance among operational fire service staff emerged. These included the physical and mental resilience borne of their training and the engagement that comes from the distinct role and culture of the fire fighter. However, there appeared other important factors such as the shift system that provides a better work–life balance and changes to policy that helped reduce the sense of entitlement to sick pay, encouraged responsibility for attendance and introduced the potential for financial penalties. We present what are to our knowledge the first detailed insights into the factors that underlie the low sickness absence rate of these public sector employees. Staff with a range of experience and rank were interviewed, reflecting the broader operational workforce. We further point out the advantages of an interviewer within the same organization, a role as occupational health advisor providing both understanding of the organization and trust within it, both of which are recommended characteristics when gathering qualitative data [14]. That saturation was reached after comparatively few interviews may be explained by consensus theory which describes how those of similar experience provide similar answers when asked about a focused topic area [15]. The response rate to the invitation to be interviewed was low but we interviewed representatives from the full range of job titles and watches as intended (Table 1).

Previously research has shown how improved physical fitness can help reduce sickness absence [16] and the physical requirements of operational staff mean that the pursuit of fitness is actively supported within the workplace. However, this is unlikely to be the sole reason for reduced sickness absence as the emphasis placed on physical fitness appeared dependent on rank and there was evidence that presenteeism might account for attendance among more senior staff. There was a similar focus on mental fortitude in the role of operational staff and despite frequent exposure to situations many might deem ‘stressful’, the operational staff we spoke to described them in terms of ‘pressure’ and ‘challenge’. This may be indicative of two things: first that masculine cultures found among employees with physically demanding jobs and ‘male gender roles’ such as the military or professional sport often view acknowledgement of stress as a weakness [17], and second that operational firefighters develop skills, qualities and attributes associated with their main role including training in how to handle complex, highly pressurised environments [18,19]. Previous research has described the benefits of matching the training of specific groups to their potential sources of stress and in particular how coping strategies can improve job satisfaction and well-being in fire fighters [20].

Engaged employees take less sickness absence [21] and characteristics of high levels of engagement that include a sense of responsibility to the organization and colleagues and a feeling of fulfilment gained from their work [22] have been observed previously in studies of firefighters [11,23] and were also described by many of those we spoke to. The importance of the quality of leadership was also referred to and previous evidence has suggested how this can encourage engagement and attendance [24]. However, tight-knit groups such as an individual watch can magnify the adverse effects of poor leadership with recent evidence that attitudes of some managers within the fire service can contribute to high attrition rates due to stress [25] and acknowledged in recent calls to remain alert to the potential for bullying in the UK fire service [26]. In acknowledging the demanding nature of their work, the positive work–life balance afforded by the four ‘rota days’ between shifts was noted and recent research has indicated the important role of this balance in reducing sickness absence [27,28].

The impact of correctly implementing a precisely worded attendance management policy has long been recognized and a number of successful policy adjustments were described by our participants. These included more robust policies to reduce the sense of entitlement and the requirement of colleagues to cover for absent team members, potentially a persuasive factor when considering the camaraderie of firefighter culture. One further policy change that appeared effective, though not without prompting concerns of presenteeism, was linking continual professional development payments to attendance. Previous research has also described the positive impact of financial incentives on work attendance [29] particularly among public sector staff, where a correlation has been observed between reduced sickness absence and increased financial penalty [30]. Here, it was notable that the most significant step appeared to be the improved training of senior staff in the implementation of these policies.

Our study has identified a number of factors that appear to contribute to continued low levels of sickness absence observed in the operational fire service. Future work might attempt to quantify some of our findings and determine further their relative importance both alone and in combination. Our qualitative work now needs underpinning with quantitative data to help determine which interventions and occupational settings would be most effective. A starting point would be to pilot interventions based on the influences we have identified in isolation across non-operational staff groups within the fire service and begin to build an evidence base of demonstrable efficacy.

**Key points**

- This is the first study of its kind to try to identify and understand the factors determining why sickness absence rates observed among operational firefighters are the lowest in the public sector.
- Though physical fitness and firefighter culture play a part, other factors came to light including the positive work–life balance afforded by fire service shift patterns and the evolution of precisely targeted policies that understand the unique nature of the operational fire service.
- The relative importance of the factors identified and how they combine should be explored further in view of the potential benefits of reducing sickness absence in other groups of public sector workers.

## Conflicts of interest

P.H. was employed directly by WMFS as a senior occupational health advisor. I.L. declares no conflicts of interest. The findings and conclusions in this work are those of the authors and do not necessarily represent the official position of WMFS.
